# Information for global mental health

**DOI:** 10.1017/gmh.2015.15

**Published:** 2015-08-19

**Authors:** A. Lora, P. Sharan

**Affiliations:** 1 Lecco Hospital of Mental Health, via dell'Eremo 9/11 Lecco 23900, Italy; 2 All India Institute of Medical Sciences, Psychiatry, New Delhi, India

**Keywords:** Mental health indicators, mental health information system, World Health Organization

## Abstract

**Background.:**

Information is needed for development of mental health (MH) services; and particularly in low- and middle-income countries (LAMICs), where the MH systems are relatively weak. World Health Organization (WHO) has worked intensively during the last 15 years for developing a strategy in the field of MH information.

**Methods.:**

The paper analyzes WHO instruments developed in this area [MH Atlas series and WHO Assessment Instrument for Mental Health Systems (WHO-AIMS)].

**Results.:**

Data from Atlas series and WHO-AIMS demonstrated that the role of information is often neglected at all the steps: from data collection and processing to analysis, dissemination and utilization, particularly in LAMIC. By implementing several sets of feasible MH indicators WHO has technically supported the improvement of the MH information systems in LAMICs.

**Conclusions.:**

In the last few years the importance of information in MH has increased and has been successfully highlighted; but a lot of work still needs to be done for achieving a minimum set of MH indicators and reliable data that can be used by both high-income countries and LAMIC.

## The role of information in mental health (MH)

Information is needed in MH, as in other health areas, but its role in MH systems is usually underestimated, not only in low- and middle-income countries (LAMICs) but also in high-income ones. High quality information is needed to monitor and to change the MH system. Without information, on the one hand, strategic choices are made without knowledge with the attendant risk of wasting resources; and on the other hand, the impact of MH policies remains unclear (without monitoring). MH systems in LAMIC are relatively weak and often not well organized, resulting in a much greater treatment gap than that found in high-income countries. To help reduce this gap, basic information is necessary about the infrastructure of the MH system, the resources available for MH, and how these resources are distributed. The risk of wasting resources is of greater salience in LAMIC settings because of scarcity of resources, e.g. the health budget allocated to MH fluctuates between 2.38% in the upper middle-income countries (UMICs) and 0.53% in the low-income ones (WHO, 2011*a*).

The paucity of data is a major challenge in LAMIC. Data are difficult to collect for several key reasons: the lack of any policy mandating the collection of health information; the absence of organizational structures to support data collection; the high proportion of health facilities over which the government has no jurisdiction [e.g. private facilities run by non-governmental organizations (NGOs)]; the shortage of trained personnel to collect health statistics; the weakness of the information technology resources and insufficient funds to support data collection.

World Health Organization (WHO) derived an intricate strategy from the World Health Report 2001 (WHO, 2001*a*) to overcome this information gap. At the policy level, WHO recommended and supported the implementation and the improvement of MH information systems in LAMICs, and helped define their structure (WHO, 2005*a*). WHO has also produced different systems of MH indicators, aimed at data collection in LAMIC in the last 14 years.

## Mental health information system (MHIS)

According to WHO (2005*a*), a MHIS is a sustainable system for collecting, processing, analyzing, disseminating, and using information about MH services and MH needs of the population it serves. Information collected within a MHIS goes through five main stages. These are:

• Collection – gathering of data;
• Processing – movement of data from the point of collection to the point of collation and preparation for analysis;
• Analysis – examination and study of the data;
• Dissemination – communication of the results of the analysis;
• Use – application of the results to improve service delivery, planning, development, and evaluation.

The last step is particularly important, but it is often neglected. In fact, MHIS is a system for action: it should exist not only for gathering data, but also for enabling well informed decision making in all aspects of the MH system. A MHIS thus seeks to establish a minimum data set (with the most essential information) that should be gathered and used.

In addition to the definition of the technical structure of a MHIS, WHO has monitored its implementation in LAMIC. This experience showed that, MHISs exist in LAMIC, although with variable levels of coverage and functioning. WHO Assessment Instrument for Mental Health Systems (WHO-AIMS) report (WHO, 2009*a*) analyzing data from 42 LAMICs, identified that about three out of four participating countries (76%) have a MH information monitoring system. This finding is consistent with the global rate of 76% reported in the Atlas 2005 (WHO, 2005*b*). However, the simple existence of a MHIS does not guarantee a well-structured and functioning information system.

WHO-AIMS report (WHO, 2009*a*) analyzed the collection of MH information within MH facilities, showing that high rates of collection were generally found in all facility types (outpatient facilities, community-based inpatient units, and mental hospitals) on basic MH information such as number of users and beds. In contrast, collection of more in-depth information, e.g. the number of involuntary admissions and restraint and seclusion, was poor across all groups of countries based on World Bank income categories and WHO regions.

MH Atlas 2011 (WHO, 2011*a*) focused on MH data that are routinely available in countries, asking the Ministry of Health for the mental information available at this level directly. At the inpatient level, about two-thirds of the countries collect MH data on admissions and days spent in mental hospitals and general hospitals, but less than one-third of the countries collect similar data from community residential facilities; also, data on service user's age, gender or diagnoses are usually available for mental hospitals and general hospitals, rarely for community residential facilities. At the outpatient level, data on patients treated in facilities, number of contacts, and service user's age, gender or diagnoses are available in more than six out of 10 countries. At primary care level, less data are available: information on service user's age, gender, and diagnoses are recorded by less than half of the countries and data related to patient contacts by a quarter. Synthesizing, relatively more MH information is available on inpatient facilities, less on outpatient ones, and relatively few data are available on community and primary care. But even this is a relatively optimistic picture, because, these data are utilized infrequently. Information, once collected, has to be analyzed, published, and disseminated. WHO-AIMS reported (WHO, 2009*a*) that, although the majority of countries have a MH monitoring system, about half of them (45%) did not publish any data, while one-third (33%) published the data without any comment and only one-fifth (21%) published reports that carried comments with the data. The lack of publication and analysis of the data is a concern. The primary purpose of a MH information collection system is not simply to gather data, but to enable decision making that will lead to service improvement. Without an intelligent dissemination of the data, enriched by comments that make the data meaningful, the likelihood of this information being used to improve service development is slim.

Concluding, the presence of a MHIS is needed but not sufficient condition for use of the available information. The deficiencies in the process that starts with data collection and ends with dissemination and use of reports become more apparent with each step of the process in LAMIC. Indeed one of the lessons learnt during the WHO-AIMS data collection was that the data are more frequently collected than supposed, but the processing, analysis, and dissemination are not planned and implemented.

## MH indicators

MH indicators are a necessary tool for using MH information. Indicators are measures which: (i) summarize information relevant to a particular phenomenon; (ii) can be used to indicate a given situation; and (iii) can therefore be used to measure change (Green, 1999). In the context of MH care, indicators are measures that summarize information relevant to the MH service and the population that it serves. At the system level, MHIS collects data that are often converted to indicators that can be used for overall service planning and policy evaluation. Indicators may be related to inputs (MH resources, e.g. human resources, facilities, financing, etc.), outputs (activities delivered by MH services) and outcomes (the effect of services on the MH of the population being served). Indicator systems usually collect information routinely on input and process/output indicators, because collecting information on outcomes in LAMIC may be complicated.

Indicators are essential to summarize large volumes of information and measure change over time. They are also a useful means of operationalizing specific policy objectives. Once indicators are identified, information systems can be designed around them. The distinction between indicators and minimum data set is that indicators are aggregates of the minimum data that have an identified denominator. For this reason, indicators are needed to determine the data set and not the other way around. The objectives of the information system should determine what data are gathered and data should not be gathered for data's sake. From this perspective an important gap in the use of MH information was the lack of agreed set of MH indicators, specially designed for data collection in LAMICs (Saxena *et al*. 2006).

To overcome this gap, WHO developed sets of MH indictors specifically designed for use in LAMIC. These systems are derived mainly from the MH Atlas series and the WHO-AIMS (WHO, 2005*c*). WHO published the first ATLAS of MH Resources around the world in 2001. Updates were produced in 2005, 2011 and 2014. The ATLAS project has become a valuable resource on global information on MH and an important tool for developing and planning MH services within countries. Feasibility was the guiding principle during the development of the Atlas indicator set; hence it contains relatively few items that are aimed at data collection in all member states, including those with rudimentary MHISs. The WHO-AIMS is an instrument designed for LAMIC that assesses key components of a MH system. The WHO-AIMS indicators analyze more issues and require collection of more data, and were implemented only in countries and regions within countries that committed themselves to the relatively more rigorous process of data collection.

The products of the MH Atlas and WHO-AIMS are different: MH Atlas publishes global figures as well as data for country groups based on World Bank income categories and WHO Regions; while the main products of WHO-AIMS were reports focused on individual countries. The report on 42 countries that adopted WHO-AIMS summarized data contained in individual country reports. These two indicators systems address different goals and cover different number of indicators, but the domains they analyze are similar: governance (MH policy and legislation, financing), MH services, activities, human resources, links with primary care and other health and social sectors, prevention and promotion, and information system. Moreover, the progresses done in one instrument automatically are reflected in the other one. Both the systems were developed with the technical assistance of the WHO Department of MH and Substance Abuse. Now we focus on a brief description of the two systems.

### WHO-AIMS

WHO-AIMS 2.2 consists of six domains: policy and legislative framework, MH services, MH in primary care, human resources, public information and links with other sectors, and monitoring and research (Saxena *et al.* 2007). These domains address the 10 recommendations of the *World Health Report 2001* through 28 facets and 155 items. All six domains need to be assessed to form a basic, yet broad, picture of a MH system, with a focus on health sector activities. WHO-AIMS provides countries with a fairly comprehensive picture of the strengths and deficiencies of their MH system, and this knowledge should facilitate improvements over time. There are structural differences between WHO-AIMS and other common indicator schemes. However, most existing monitoring instruments focused more narrowly on the psychiatric services sector. WHO-AIMS explored areas like the link with other sectors and governance of the MH sector that are essential to have a global picture of the MH system.

WHO-AIMS primarily consists of input indicators (resources that are used to develop or modify systems and services) and process indicators (service utilization, quality of services, and programs). It is important to recognize that WHO-AIMS contain a number of ordinal rating scales, because precise data are difficult to collect in some settings. In this case respondents are instructed to provide precise data if these are available, and if data are not available, to provide their best estimate on the ordinal scale based on other information or a data source for example, after consulting key informants. In addition, in terms of content, WHO-AIMS contains a number of items that measure important MH resources in LAMIC that may not be as critical as in high-income countries; e.g. items addressing traditional healers and non-professional primary health care workers. Finally, in terms of data collection, WHO-AIMS requires collection of information from all relevant organizations at all levels of the health system, encouraging countries to locate existing data that have not been transmitted or analyzed.

The purpose of the WHO-AIMS project is to enable countries to collect baseline information on MH that can be used to strengthen MH systems. The majority of participating countries were able to collect and report data for most of the WHO-AIMS indicators, suggesting that a systematic, quantitative assessment of MH systems in LAMICs is possible. Furthermore focal points in 42 countries that completed the WHO-AIMS assessment were re-contacted and asked to respond to five questions to determine whether these countries had utilized the information collected through WHO-AIMS to strengthen their MH systems (WHO, 2005*c*): (1) have WHO-AIMS results been presented in a national workshop, (2) have WHO-AIMS results been used to develop or revise a MH policy of plan, (3) have WHO-AIMS results been used for another planning purpose, (4) has a scientific article been published using WHO-AIMS results, and (5) has WHO-AIMS been used to improve the MH information monitoring system.

As summarized in Table 1 follow up activity occurred in the majority of countries that completed a WHO-AIMS assessment. Thirty-one out of 42 countries (74%) have presented WHO-AIMS results in a national workshop to relevant stakeholders and almost half of the countries have used WHO-AIMS to develop or revise a MH policy or plan. An additional eight countries (19%) are either in the process of or are planning to use WHO-AIMS for this purpose. Fifty-five per cent of countries have used WHO-AIMS for some other planning purpose and 24% have published a scientific article using WHO-AIMS results. Finally, 12 out of 42 (29%) countries have used WHO-AIMS indicators to improve their MH monitoring system.

**Table 1.** Impact of the WHO-AIMS Assessments (n = 42)

	Presented in national workshop	Used to develop policy or plan	Used for other planning purpose	Scientific article published	Used to improve mental health monitoring system
Yes	31 (74%)	18 (43%)	23 (55%)	10 (24%)	12 (29%)
No	10 (24%)	16 (38%)	17 (41%)	24 (57%)	24 (57%)
Planned/in progress	1 (1%)	8 (19%)	2 (5%)	7 (17%)	6 (14%)

As can be seen from the sample responses, WHO-AIMS has been instrumental in developing or revising MH policies or plans in a number of countries. Moreover, selected responses to the question ‘has WHO-AIMS been used for any other planning purpose’ indicate that WHO-AIMS results have been used in multiple ways to inform MH planning. Overall, the results of the follow-up survey to countries that completed a WHO-AIMS assessment suggest that these countries used the information for improving MH systems.

### MH Atlas

Project Atlas was launched in 2000 by WHO, as an effort to catalogue and analyze the country-level data on resources available to manage mental and behavioural disorders. Publications based on the Project (WHO, 2001*b*, 2005*b*, 2011*a*) presented data on various parameters related to MH resources and services, namely national policies and legislation on MH; budgetary issues; MH facilities at all levels of care (primary, community-based, general hospitals, and mental hospitals); human resources; therapeutic psychotropic medication, and MH reporting systems. Information for this project was obtained from the focal points for MH in the Ministries of Health in each WHO Member State, Associate Member, and Area through WHO Regional Offices.

It is critical to monitor progress as even small improvements in the global situation could lead to significant quality-of-life benefits, as well as human rights and economic improvements worldwide. The 2011 Atlas differs from the previous work, in that, its methodology makes use of indicators developed in WHO-AIMS (Table 2). This methodological improvement, involving more labour-intensive data collection, and extensive review procedures, has led a more standardized reporting of indicators. Despite differences in methodology and indicators, taken together, the existence of the MH Atlas at three time points, alongside comprehensive WHO-AIMS country reports, allows for a broader view of how resources for MH have developed over the last 10 years at a global level.

**Table 2.** Indicators used in Atlas 2001/2005 and 2011

Atlas 2001/ 2005	Atlas 2011
*Governance*	
• Presence of mental health (MH) policy • Presence of MH legislation • Presence of substance abuse policy • Presence of national MH programme • Presence of disability benefits	• Presence of dedicated MH policy • Presence of dedicated MH legislation – • Presence of MH plan –
*Financing*	
• Presence of specified budget for MH • Percentage of health budget allocated to MH - • Most common method of financing MH care (tax based, out-of-pocket, social insurance, private insurance, and others)	• MH expenditures *per capita* – • Percentage of health budget allocated to MH • Percentage of MH expenditures on mental hospitals –
*MH care delivery*	
(i) Primary health care (PHC)
• Presence of MH care in primary care • Presence of facilities for management of severe mental disorders in primary care – • Presence of training facilities for primary care personnel in MH – –	– • Regulation on prescription of medicines for mental and behavioural disorders by PHC staff – • Policy enabling PHC nurses to independently diagnose and treat mental disorders within the primary care system • Presence of in-service training on MH in PHC • Availability of manuals on the management and treatment of mental disorders in the majority of PHC settings • Referral procedures between primary care and secondary/tertiary care
(ii) MH facilities
• Presence of community care for MH – – – – – – • Rate of psychiatric beds in general hospitals per 100 000 population – – – – • Presence of mental hospitals – • Rate of beds in mental hospitals per 100 000 population –	– • Rate of MH outpatient facilities per 100 000 population • Annual rate of outpatients per 100 000 population • Presence of day treatment facilities • Rate of day treatment facilities per 100 000 population • Annual rate of persons per 100 000 population treated in MH day treatment facilities • Presence of psychiatric wards in general hospitals • Rate of psychiatric beds in general hospitals per 100 000 population • Annual rate of admissions to psychiatric beds in general hospitals per 100 000 population • Presence of community residential facilities • Rate of community residential facilities per 100 000 population rate of community residential facility beds per 100 000 population • Rate of persons staying in community residential facilities per 100 000 population • Presence of mental hospitals • Rate of mental hospitals per 100 000 population • Rate of beds in mental hospitals per 100 000 population • Annual rate of admissions per 100 000 population to mental hospitals
(iii) Service dimensions
– – – • Rate of psychiatric beds *per 10,000 population* • Percentage of beds for psychiatric patients by facility type (mental hospitals, general hospitals, and community residential facilities)	• Length of admissions to mental hospitals • Routine follow-up community care at MH facilities • Provision of psychosocial interventions by a majority of MH facilities • Rate of beds per 100,000 population • Percentage of beds for psychiatric patients by facility type (mental hospitals, general hospitals, and community residential facilities)
*Human resources*	
– – – • Rate of human resources per 100 000 population working in the MH sector (psychiatrists, other medical doctors, nurses, psychologists, and social workers) • Comparison of mean number of psychiatrists with psychiatric nurses *per 100 000 population* • Rate of human resources per 100 000 population working in the MH sector (Neurologists, Neurosurgeons) – –	• Rate of human resources graduates in the past academic year per 100 000 population (psychiatrists, other medical doctors, nurses, psychologists, social workers, and occupational therapists) • Percentage of training devoted to MH-related subjects in graduate programmes related to MH • Number of human resources (per 100 000 population) working in the MH sector • Rate of human resources per 100 000 population working in the MH sector (psychiatrists, other medical doctors, nurses, psychologists, social workers, and occupational therapists) • National MH governance – – • Presence and involvement of user and family associations
*Medicines for mental and behavioural disorders*	
• Presence of therapeutic drug policy/essential list of drugs • Availability of therapeutic drugs in primary care • Availability of three essential therapeutic psychotropic drugs at primary care level (phenytoin, amitriptyline, and chlorpromazine) – – • Annual expenditure of treatment with psychotropic drugs per person (phenytoin 300 mg/day, amitriptyline 150 mg/day), and chlorpromazine 400 mg/day)	– – – • Annual expenditures on medicines for mental and behavioural disorders per 100 000 population • Annual expenditures on medicines for mental and behavioural disorders per 100 000 population (mood stabilizers, antipsychotics, anxiolytics, and antidepressants)
*Information systems*	
• Presence of MH reporting system – • Presence of epidemiological study or data collection system in MH	– • MH data routinely available in the ministry of health on (persons treated, service users’ age and gender, service users’ diagnosis –
*MH programmes for special populations*	
• Presence of MH programme for special populations (children, elderly persons, indigenous people, disaster affected populations, refugees, and minority groups) • Presence of NGOs in MH	– –

The MH Atlas 2005 data showed that across the world, annual MH reporting systems exist in 75.8% of countries, though their quality and coverage vary enormously. About three-fifths of low-income and four-fifths of middle- and high-income countries reported that they had these systems. Compared with 2001 data there was an increase (+9.3%) in the number of countries with such systems in lower middle-income countries (LMICs).

MH Atlas-2011 data show that data collection from primary care and residential care facilities was conducted only in one-third of reporting countries. For general hospitals and mental hospitals, data were available from more than half of the respondents. Data on number of admissions, days spent in hospitals, and diagnosis, were available in excess of data on age, gender, etc. Regional imbalances and differences across groups of countries based on income categories persist. In terms of dissemination, approximately four countries out of every 10 produce a report focused on MH activities. However, such publications are less frequent in low and middle income countries. This result underscores the work that still remains to be done, as good governance requires the analysis and dissemination of information, as well as the inclusion of additional stakeholders to promote accountability.

Formal assessment of impact of Project Atlas has not been conducted by WHO or other agencies. In relation to resources, MH Atlas 2011 indicates that little has changed in the allocation of resources for MH care during the last 10 years, particularly in LAMICs. However, the fact that the vast majority (>76%) of MH policy and plan documents have been approved or updated since 2005 and the majority (51%) of legislative documents since 2001, and that a clear trend was found for a decrease in the rate of mental hospital beds in all income groups of countries and five of six WHO regions between 2005 and 2011, suggests a positive policy impact of Atlas data generated since 2001. Over the last decade, the WHO Mental Health Gap Action Programme (mhGAP) (2008), and related initiatives such as *The Lancet*'s Series on Global Mental Health (2007 and 2011), as well as initiatives such as the Movement for Global Mental Health and the World Federation for Mental Health's ‘Great Push for Mental Health’ have used Atlas data for advocacy purposes.

These data emphasize the urgent need to enhance resources devoted to MH information, especially in LAMICs. It is hoped that the recent focus on providing such indicators and regular monitoring exercises like the MH Atlas and the WHO-AIMS project would help policy-makers at the national and international levels to evaluate and improve their working, towards providing affordable and adequate MH care facilities that are easily available to all potential users.

### The MH action plan 2013–2020 and MH atlas 2014

In May 2013, the 66th World Health Assembly adopted the Comprehensive Mental Health Action Plan 2013–2020. Global targets have been established in order to measure collective action and achievement by WHO's member states towards the overall goal and objectives of the Action Plan. A set of core MH indicators have been developed to monitor progress in relation to these targets and other critical aspects of MH system development (Table 3). It is expected that these core MH indicators will be collated and reported every 2 years, starting with a baseline assessment for the year 2014. Collation of core MH indicators on a regular basis will be embedded within an updated version of WHO's MH Atlas. Subsequent to this baseline data collection in 2014, a MH ATLAS survey will be sent to country focal points every 2 years so that progress towards meeting the targets of the Action Plan can be measured over time (until 2020).

**Table 3.** Mental health (MH) action plan targets and indicators

Action plan objectives	Action plan targets	Action plan indicators	Service development indicators
*Objective 1*: To strengthen effective leadership and governance for MH	*Target 1.1: 80% of countries will have developed or updated their policies or plans for MH in line with international and regional human rights instruments (by the year 2020).*	Existence of a national policy/plan for MH that is in line with international and regional human rights instruments	*Financial resources*: Government health expenditure on MH
			*Human resources*: Number of MH workers
	*Target 1.2: 50% of countries will have developed or updated their law for MH in line with international and regional human rights instruments (by the year 2020).*	Existence of a national law covering MH that is in line with international and regional human rights instruments	*Capacity building*: Number and proportion of primary care staff trained in MH
*Objective 2*: To provide comprehensive, integrated and responsive MH, and social care services in community-based settings	*Target 2: Service coverage for severe mental disorders will have increased by 20% (by the year 2020).*	Number and proportion of persons with a severe mental disorder who received MH care in the last year	*Stakeholder involvement*: Participation of associations of persons with mental disorders and family members in service planning and development
			*Service availability*: Number of MH care facilities at different levels of service delivery
*Objective 3*: To implement strategies for promotion and prevention in MH	*Target 3.1: 80% of countries will have at least two functioning national, multisectoral MH promotion and prevention programmes (by the year 2020)*	Functioning programmes of multisectoral MH promotion and prevention in existence	*Inpatient care*: Number and proportion of admissions for severe mental disorders to inpatient MH facilities that a) exceed 1 year, and b) are involuntary
	*Target 3.2: The rate of suicide in countries will be reduced by 10% (by the year 2020).*	Number of suicide deaths per year	*Service continuity*: Number of persons with a severe mental disorder discharged from a mental or general hospital in the last year who were followed up within 1 month by community-based health services
*Objective 4*: To strengthen information systems, evidence, and research for mental health	*Target 4: 80% of countries will be routinely collecting and reporting at least a core set of MH indicators every 2 years through their national health and social information systems (by the year 2020).*	Core set of MH indicators routinely collected and reported every 2 years	
			*Social support*: Number of persons with a severe mental disorder who receive disability payments or income support

The Action Plan indicator system explores some traditional areas covered by WHO-AIMS and MH Atlases (i.e. governance, financing, human resources, MH activities, information system, etc.), but often with addition of new insight. For example, the existence of a mental policy and legislation is enriched with the addition of enquiry on compliance with international human rights instruments. Also, the involvement of stakeholders (e.g. family and users associations) in the formulation and implementation of MH policies, laws and services at the national level is analyzed in depth. In terms of MH activities, specific attention is paid to service coverage, e.g. an item read ‘s*ervice coverage for severe mental disorders will have increased by 20% (by the year 2020)*’. Indicators focused on suicide rates and prevention activities have also been introduced. The Atlas 2014 indicators represent a step forward, as elements of quality assessment have been added (e.g. coverage, continuity of care). But the indicator-set also represents a challenge because of the difficulties that many countries met in responding to these items. However, the long term (over the next 10 years) demand for these data may support the improvement of data quality.

## An overview of the results from the atlases and WHO-AIMS data collections

In this section an overview is presented of the main results provided from the MH Atlas 2011 and WHO-AIMS project. On the one hand data derived from the WHO's MH Atlas Project 2011 (WHO, 2011*a*; Morris *et al*. 2013) provide the latest global estimates on available resources for the treatment of neuropsychiatric disorders covering 98% of the world's population. On the other hand report based on WHO-AIMS methodology (WHO, 2009*a*), details the level of resources available in the MH systems of 42 LAMICs, and provides in depth analysis of these MH systems, regarding how those resources are organized and utilized. Thus, data on efficiency, access, equity, linkages with other sectors, and respect for human rights are reported as well.

### ATLAS 2011

Resources are defined in terms of governance, financing, MH care delivery, human resources, essential medicines, and information systems.

#### Governance

Results indicate that 60% of countries have a dedicated MH policy; 71% possess a MH plan; and 59% report having dedicated MH legislation. The vast majority of policy and plan documents have been approved or updated since 2005 and the vast majority of legislative documents since 2001. A much higher percentage of high income countries report having a policy, plan, and legislation than low income countries (LICs).

#### Financing

The median MH expenditures *per capita* is US$1.63, with large variation among income groups, ranging from US$0.20 in LICs to US$44.84 in high income countries. Globally, 67% of financial resources are directed towards mental hospitals.

#### MH services

The global median number of facilities per 100 000 population were 0.61 outpatient facilities, 0.05 day treatment facilities, 0.01 community residential facilities, and 0.04 mental hospitals.

Overall, mental hospitals still represent the primary mode of inpatient service: they are present in 80% of countries. There are 7.04 psychiatric beds per 100 000 population in mental hospitals in comparison with 1.4 psychiatric beds per 100 000 population in general hospitals. Globally, the median rate of admissions to mental hospitals is 39.3 per 100 000, while that in psychiatric wards in general hospitals is 24.2 per 100 000 population. Higher income countries typically have more facilities and higher admission/utilization rates. As far as the patients admitted in mental hospitals are concerned, the significant majority (77%) of these individuals remain admitted for less than 1 year. However, this also implies that almost a quarter of people admitted to mental hospitals remain there longer than a year after admission, becoming long-term patients. The process of deinstitutionalization can be observed by comparing data from 2005 and 2011. Globally, the median decrease in mental hospital beds was −0.11 per 100 000 population, indicating that the majority of countries reduced their rate of mental hospital beds over this period. This would imply that, in a country with a population of 10 million people, there would be an expected decrease of 11 beds over this period. Beds in mental hospitals decreased mainly in high income (−0.43) and UMICs (−0.90), as compared with lower-middle (−0.14) and LICs (−0.01), which is understandable considering the low bed to population ratios in lower-middle and LICs.

**Fig. 1.**
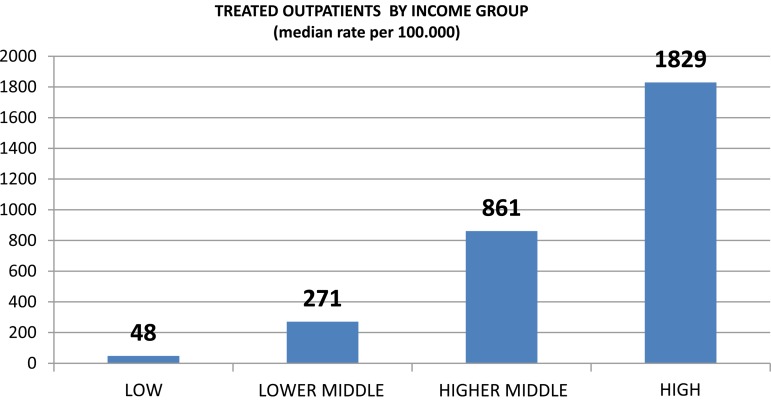
Treated outpatients by income group.

The median annual rate of service users treated in outpatient clinics per 100 000 population is 384, with substantial variability by country income level (Fig. 1). Accessibility, in terms of the rate of treated outpatients, is six times greater in high-income countries as compared with LMICs, and 38 times higher than in LICs. Similarly, the rate of outpatient contacts in high-income countries is 10 times higher than in LMICs and 50 times higher than in LICs. Only 32% of countries provide follow-up care in a majority of facilities. This figure varies across income classifications; 7% of low income, 29% of lower-middle income, 39% of upper-middle income, and 45% of high-income countries provide follow-up care at a majority of facilities. Similarly, only 44% of countries provide psychosocial interventions at a majority of facilities, a figure which also varies by income classification; 14% of low-income, 34% of lower-middle income, 61% of upper-middle income, and 59% of high-income countries provide psychosocial care at a majority of facilities.

#### Human resources

**Fig. 2.**
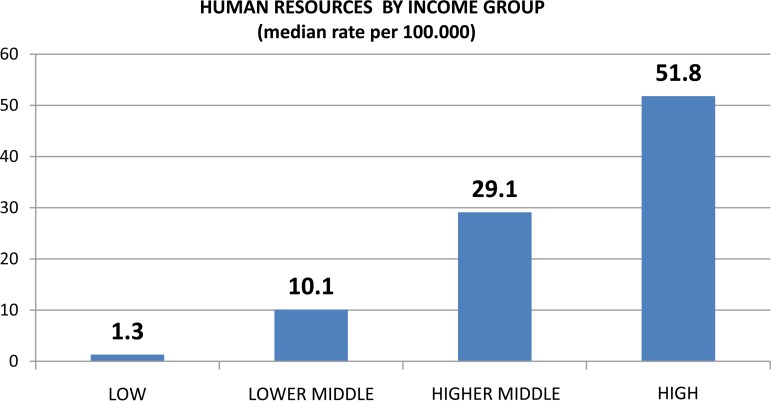
Human resources by income group.

There is a clear pattern whereby greater rates of human resources are observed in higher income countries (Fig. 2). Globally, nurses represent the most prevalent professional group working in the MH sector. The median rate of nurses in this sector (5.8 per 100 000 population) is greater than the rate of all other human resources groups combined. For all human resources, there is a clear pattern whereby greater rates of human resources are observed in higher income countries. For example, there is a median rate of 0.05 psychiatrists (per 100 000 population) in LICs, 0.54 in LMICs, 2.03 in UMICs, and 8.59 in high-income countries. User and family associations are present in about two-thirds of the countries (64 and 62% of countries, respectively), with greater representation in higher income countries. User associations are more prevalent in higher income countries – in 83% of high-income countries *v.* 49% of LICs – as are family associations, which are present in 80% of high-income countries and 39% of LICs.

#### Medicines for mental and behavioural disorders

Globally, the estimated median expenditure on medicines for mental and behavioural disorders is US$6.81 per person per year. However, the true figure is likely to be substantially lower; only 49 of 184 countries (27%) reported these data, and respondents were disproportionally high-income countries.

### WHO-AIMS

Results indicate that MH systems in LAMICs are providing care to only a small proportion of all those who need it. The median treated prevalence rate of 0.67% of the population per year in this study is a small fraction of what would be expected based on community epidemiological studies. The corresponding treated prevalence rate for children and adolescents is even lower. While it is estimated that approximately one in 20 children has a severe mental disorder, the median treated prevalence rate of 0.16% of the child population per year reported in this study suggests that the overwhelming majority of children and adolescents with severe mental disorders in LAMICs receive no treatment.

Results confirm that MH resources in LAMICs are scarce, inequitably distributed and inefficiently used. The median number of MH professionals is 6 per 100 000 population, and MH spending *per capita* is US$0.30 – a mere fraction of the US$3–4 suggested by WHO (2006) for a basic package of care. The dearth of resources is particularly pronounced in LICs, resulting in a wide gap between LICs and UMICs: MH spending *per capita* is 70 times higher in the reporting UMICs than in reporting LICs, there are 24 times more beds per 100 000 population in community-based inpatient units, 10 times more community outpatient contacts, and 8 times more MH staff in UMICs than in LICs.

Available resources are inefficiently used: eight psychiatric beds in 10 are located in mental hospitals, yet these facilities provide care for only 7% of all services users. These facilities also consume most or a disproportionately large share of the available finances. The median proportion of MH finances spent on mental hospitals was 80%, thus depriving community services of much needed funds. In addition to being scarce and inefficiently used, resources for MH systems tend to be inequitably distributed. The vast majority of MH beds and staff are concentrated in the largest cities. Insufficient, inequitable, and inefficient use of resources greatly impede access to MH care: results indicate that only one in three people with schizophrenia are currently receiving treatment. On a more positive note, the number of beds in mental hospitals in middle-income countries is decreasing in favour of community care, which is more cost effective and has less scope for human rights abuses. However, for the majority of the participating countries, the transition to community care is slow: the number of beds in mental hospitals is not decreasing in LICs, and in LMICs, where inpatient care is still predominant. Overall, there are still only 0.7 outpatient contacts for every day spent in inpatient care. Moreover, day treatment facilities and community residential facilities are scarce across all countries, but particularly among LICs and LMICs.

The data suggest that connections between MH and other relevant components of the health system as well as non-health sectors are weak. Although the majority of countries reported formal collaboration between MH care and primary health care (PHC) departments, in assessing MH care activities within the primary care system, the data suggest that there is little, integration of MH into PHC. For example, psychotropic medicines and assessment and treatment protocols are not widely available, and few PHC clinics make regular referrals to a higher level of care.

WHO-AIMS data show that in a number of countries there is scant attention to human rights of service users. MH legislation exists in only half of the 42 reporting countries, human rights inspections and training are infrequent, and collection of data on involuntary admissions and physical restraint and seclusion is limited. Moreover, user and family associations, which are key allies in advocacy for the care and rights of people with mental disorders, are absent in approximately half of the countries.

## The way forward

According to WHO, information is one of the six building blocks of the MH systems (WHO, 2007, 2009*a*, *b*). Starting from the World Health Report 2001 (WHO, 2001*a*), WHO is committed to the development and strengthening of the use of MH information in LAMIC, specifying the characteristic of MH information systems and defining systems of indicators tailored on the needs of these countries. The WHO-AIMS and Atlases assessment for many countries was the first time that comprehensive information on their MH system has been gathered and disseminated. Not only do the data provided baseline information that can be used to develop plans to strengthen or scale up services, but also the process of collecting the data has brought together key stakeholders within many countries.

The WHO hopes to bring about a system change through monitoring, where information is not gathered sporadically, but rather produced routinely inside the MH system.

Nevertheless, despite these actions, many problems still remain. The scarcity of resources devoted to MH information, reflects the general scarcity of resources devoted to MH. This causes weaknesses of information technology network dedicated to MH, poor integration with general health information systems, and lack of surveys addressing MH needs. But it is not only a matter of resources. The phrase ‘what gets measured gets done’ still goes unheeded by most MH professionals working in LAMIC as well as high-income countries. A ‘data oriented’ culture is lacking among MH professionals and managers, as is training on monitoring and evaluating MH services. Concerning data collection, information on service delivery, and human resources (which are commonly included in information systems) should be covered more adequately, guaranteeing a minimum data set at the facility level. Similarly, more needs to be done in other areas of great interest for MH systems, like primary care or social services, for which information is scarce not only in LAMIC but also in high-income countries. More information is needed to improve human rights in MH facilities: e.g. data on compulsory admissions, and restraints and seclusions. Data transmission is often incomplete and it is frequently found that information was collected but was not transmitted to Ministry of Health. The dissemination of the data is frequently undervalued and many data still remain unanalyzed and unutilized. The use of information for advocacy and planning, still remains undervalued and scarcely implemented not only in LAMICs but also in high-income countries.

In the last years the role and importance of information in MH has been successfully highlighted, but a lot of work still remains to be done for achieving a minimum set of MH data and indicators that are collected homogenously and utilized for changing MH systems. WHO on the basis of the experience acquired through the MH Atlas and WHO-AIMS data collection exercises has identified a framework of internationally recognized and accepted MH indicators, that could be useful for monitoring MH Action Plan and that could define a minimum data set of items feasible for use in LAMIC.

However, implementation of mental information system in LAMICS also have to deal with other broad challenges Ryan *et al*. (2015), starting from their work for implementing a state-level MHIS in Nigeria identified three such challenges. First, the challenge of collecting data from primary care (a challenge also in high-income countries) consequent to the decentralization of services being recognized as a fundamental strategy for scaling up MH care. Second is the integration of parallel information systems, deriving from public and private services, given that the MH system in LAMICs is a patchwork of public and private initiatives. Third, the lack of adequate resources for Information Technology and information system, and human resources needed to manage the system.

Some international projects are working towards overcoming some of these difficulties and for implementing a more efficient MH information system, as a part of a wider improvement of the MH systems in LAMICs.

As a component of the *Mental Health and Poverty Project* (MHaPP), a 5-year project on MH policy development and implementation in four African countries; new MH information systems were developed and trialled over a 1-year pilot project period using the WHO model. In South Africa, new MH indicators were integrated into the national District Health Information System in pilot sites in two provinces; and in Ghana, the three psychiatric hospitals standardized their methods of collecting data. In both countries, MH information is now available for policy, planning, and management decisions.

The *Emerging mental health systems in LAMICs* (EMERALD) programme funded by the European Commission aims to identify key health system barriers to, and solutions for, the scaled-up delivery of MH services in six LAMICs in Asia and Africa and by doing so improve MH outcomes in a fair and efficient way. Specifically, the EMERALD Project aims to develop, use, and monitor indicators of MH service coverage and system performance.

The *Programme for Improvement of Mental health care* (PRIME) is operating in five LMICs (Ethiopia, India, Nepal, South Africa, and Uganda) to provide evidence to support the implementation and scale-up of MH care in primary care and maternal health care settings. Starting from the consideration that a comprehensive information about treatment coverage for mental, neurological, and substance use (MNS) disorders was lacking across all sites, the project is working towards improving the recording and reporting of service utilization and for identifying optimal service utilization indicators for MH care integrated into PHC in LMICs.

At country level, the WHO work in sustaining the role of information in LAMICs has been useful to push the role of information when a country is strengthening their MH system. China is a good example of this process. Promoted by the requirement by its MH Law, China (Zhou & Xiao, 2015) is working towards expanding the existing public MH surveillance from its focus on psychosis, to the inclusion of more categories of MH problems, like depression and substance-related and addictive disorders, and a greater coverage of MH-related events.

According to a WHO document on information systems (WHO, 2011*b*) though paper-based systems continue to dominate, there is increasing evidence from local studies that careful system design with innovation through information and communication technology is providing some solutions. For instance the monitoring of service availability is gradually improving through the development and updating of facility databases and increased use of geographic information systems.

Service delivery and human resources, are building blocks of the MH system, hence, a minimum data set of these items should be collected, starting from the facility level. However a major weakness of the MH information systems in LAMICs is often poorly functioning health facility reporting system that continues to be plagued by data quality problems. In 2013, WHO launched Service Availability and Readiness Assessment (SARA) (WHO, 2013) to conduct a systematic assessment of health facility service delivery. The objective of the survey is to generate reliable and regular information on service delivery including service availability, such as the availability of key human and infrastructure resources, and on the readiness of health facilities to provide basic health-care interventions relating to family planning, child health services, basic and comprehensive obstetric care, HIV/AIDS, tuberculosis, malaria, and non-communicable diseases. The SARA survey generates a set of tracer indicators to assess and monitor availability of health services and readiness to provide services. Although SARA does not include tracer indicators on MH, its methodology may be interesting for implementing data collection of basic MH indicators at facility level.

In recognition of the role of MHIS for guiding system development, WHO's MH Action Plan 2013–2020 has included strengthening of information systems, evidence and research for MH as a key strategy towards setting new directions for MH. The MH action plan also contains key indicators that member countries may use to monitor the achievement of goals. It is hoped that the recent focus on providing such indicators and regular monitoring exercise, like the MH Atlas and the WHO-AIMS project, would help policy-makers at the national and international levels to strengthen the information infrastructure and through a better information to provide affordable and adequate MH care.
